# Hypoxemia in COVID-19: cerebral oximetry should be explored as a warning indicator for mechanically ventilated adults with COVID-19

**DOI:** 10.1186/s12931-020-01530-w

**Published:** 2020-10-09

**Authors:** Marco Ferrari, Valentina Quaresima

**Affiliations:** Department of Life, Health and Environmental Sciences, University of L’Aquila, Via Vetoio, 67100 L’Aquila, Italy

**Keywords:** COVID-19, SARS-CoV-2, Respiratory failure, Hypoxemia, Cerebral oximetry, NIRS, Gas exchange

**Letter to the editor**

We read with interest the review on the pathophysiology of ‘happy’ hypoxemia in COVID-19 by Dhont et al. published in *BMC Respiratory Research* [1]. This exhaustive review, describing the pathophysiological abnormalities in COVID-19 that might explain the disconnect between the severity of hypoxemia and the relatively mild respiratory discomfort reported by the patients, aims at improving decision-making and management among the physicians treating COVID-19. Very recent articles and reviews on the neurological manifestations of COVID-19 report patients with severe COVID-19 at risk for multifocal microvascular hemorrhagic and ischemic lesions [2–7]. Therefore, it would be very valuable to monitor the brain oxygenation state in mechanically ventilated patients with COVID-19.

In the last decades, brain oxygenation has been successfully monitored noninvasively and transcranially in the operative room and in the intensive care unit by commercial near-infrared spectroscopy (NIRS) brain oximeters [8–11]. These oximeters provide the intensivists with a continuous measure of the prefrontal cortex oxyhemoglobin saturation (ScO_2_, %). Unlike conventional fingertip pulse oximetry, ScO_2_ does not rely on a pulsating flow, and reflects the balance between oxygen supply and demand in the arteriolar, capillary and venular beds of the brain cortex underlying the prefrontal area over the sensor is located. Detailed cerebral oximetry guidelines have recently been drawn up by the Japanese Society of Cardiovascular Anesthesiologists [12].

To the best of our knowledge, so far the use of cerebral oximetry on ventilated COVID-19 patients has never been reported. In this framework, we suggest that the ScO_2_ monitoring in these patients might serve as an “early warning indicator” of the decreased brain oxygen delivery. The ScO_2_ data can be utilized to optimize cerebral oxygen supply and demand, inversing the decreased cerebral perfusion and/or preventing protracted brain ischemia.

Interestingly, cortical oximetry is utilized in an ongoing clinical trial of the Hvidovre University Hospital (Denmark) aiming to examine whether ScO_2_ could be a more useful parameter than peripheral arterial oxygen saturation, measured by fingertip pulse oximetry, to guide clinical titration of permissive hypoxemia in COVID-19 acute respiratory distress syndrome patients [13].

## Data Availability

Not applicable.

## Abbreviations

NIRS: Near-infrared spectroscopy
ScO_2_: Oxyhemoglobin saturation

## References

[1] Dhont S, Derom E, Van Braeckel E, Depuydt P, Lambrecht BN (2020). The pathophysiology of ‘happy’ hypoxemia in COVID-19. Respir Res.

[2] Ahmad I, Rathore FA (2020). Neurological manifestations and complications of COVID-19: a literature review. J Clin Neurosci.

[3] Gupta A, Madhavan MV, Sehgal K (2020). Extrapulmonary manifestations of COVID-19. Nat Med.

[4] Jaunmuktane Z, Mahadeva U, Green A (2020). Microvascular injury and hypoxic damage: emerging neuropathological signatures in COVID-19. Acta Neuropathol.

[5] Kremer S, et al. Brain MRI findings in severe COVID-19: a retrospective observational study. Radiology. 2020:202222. 10.1148/radiol.2020202222. 10.1148/radiol.2020202222 PMC7301613 32544034

[6] Nepal G, Rehrig JH, Shrestha GS, Shing YK, Yadav JK, Ojha R (2020). Neurological manifestations of COVID-19: a systematic review. Crit Care.

[7] Whittaker A, Anson M, Harky A (2020). Neurological manifestations of COVID-19: a systematic review and current update. Acta Neurol Scand.

[8] Benni PB, MacLeod D, Ikeda K, Lin HM (2018). A validation method for near-infrared spectroscopy based tissue oximeters for cerebral and somatic tissue oxygen saturation measurements. J Clin Monit Comput.

[9] Eyeington CT, Ancona P, Osawa EA, Cutuli SL, Eastwood GM, Bellomo R (2019). Modern technology-derived normative values for cerebral tissue oxygen saturation in adults. Anaesth Intensive Care.

[10] Ferrari M, Quaresima V (2012). Review: near infrared brain and muscle oximetry: from the discovery to current applications. J Near Infrared Spectr.

[11] Tosh W, Patteril M (2016). Cerebral oximetry. BJA Educ.

[12] Yoshitani K, Kawaguchi M, Ishida K, Maekawa K, Miyawaki H, Tanaka S (2019). Guidelines for the use of cerebral oximetry by near-infrared spectroscopy in cardiovascular anesthesia: a report by the cerebrospinal Division of the Academic Committee of the Japanese Society of Cardiovascular Anesthesiologists (JSCVA). J Anesth.

[13] Clinical Trial: effects of cardiovascular and pulmonary optimization on cerebral oxygenation in COVID-19 patients with severe ARDS (NIRS-COV). ClinicalTrials.gov Identifier: NCT04392089. https://clinicaltrials.gov/ct2/show/NCT04392089. Accessed 24 Aug 2020.

